# Socioeconomic conditions and number of pain sites in women

**DOI:** 10.1186/1472-6874-12-7

**Published:** 2012-03-29

**Authors:** Toril Rannestad, Finn Egil Skjeldestad

**Affiliations:** 1 Research Centre for Health Promotion and Resources HiST/NTNU, Sør-Trøndelag, University College, Faculty of Nursing, N-7004 Trondheim, Norway; 2 Department of Infectious Disease Epidemiology, Norwegian Institute of Public Health, PO Box 4404, Nydalen 0403, Oslo, Norway; 3 Women's Health and Perinatology Research Group, Department of Clinical Medicine, Faculty of Health Sciences, University of Tromso, N-9038 Tromso, Norway

**Keywords:** Socioeconomic conditions, Number of pain sites/locations, Women, Co-morbidity, Cancer survivors

## Abstract

**Background:**

Women in deprived socioeconomic situations run a high pain risk. Although number of pain sites (NPS) is considered highly relevant in pain assessment, little is known regarding the relationship between socioeconomic conditions and NPS.

**Methods:**

The study population comprised 653 women; 160 recurrence-free long-term gynecological cancer survivors, and 493 women selected at random from the general population. Demographic characteristics and co-morbidity over the past 12 months were assessed. Socioeconomic conditions were measured by Socioeconomic Condition Index (SCI), comprising education, employment status, income, ability to pay bills, self-perceived health, and satisfaction with number of close friends. Main outcome measure NPS was recorded using a body outline diagram indicating where the respondents had experienced pain during the past week. Chi-square test and forward stepwise logistic regression were applied.

**Results and Conclusion:**

There were only minor differences in SCI scores between women with 0, 1-2 or 3 NPS. Four or more NPS was associated with younger age, higher BMI and low SCI. After adjustment for age, BMI and co-morbidity, we found a strong association between low SCI scores and four or more NPS, indicating that there is a threshold in the NPS count for when socioeconomic determinants are associated to NPS in women.

## Background

Living in deprived socioeconomic conditions is associated with higher prevalence of health complaints [1,2], like generalized [3], musculoskeletal [4], chronic non-malignant [5] and complex/frequent/intensive pain [6]. The most frequent measures of self-reported pain are frequency, severity, and specific pain locations. During the recent years number of pain sites (NPS) is regarded as a better parameter in pain assessment [7] and may be more important than actual sites in determining the impact on health [8] and functioning [9]. Females endorse a larger NPS than males [7,10-12], and high NPS is frequently reported around middle age [7,12,13]. Increasing NPS is related to impaired health status [10,12], and poorer general [9,14], as well as physical, psychological and social functioning outcomes [15]. However, little is known regarding the association between socioeconomic conditions and NPS. The aim of this study was to explore the relationship between socioeconomic conditions and NPS in women.

## Materials and methods

### Study population

The study population comprised recurrence-free long-term gynecological cancer survivors and women from the general population. From 1987 through 1996, 1171 primary patients of cervical, corpus and ovarian cancer were treated at St. Olav's Hospital, Trondheim, which represent all gynecological cancer patients from the middle part of Norway. In May 2003 we examined survival and recurrence status. Women aged 30-75 years and without recurrence of disease, were eligible for participation in this cross-sectional study - in total 369 cases. For each survivor we selected four age-matched women as controls, living in the same county, at random from the population census. We failed to reach 50 cases due to invalid mailing addresses. Thus, the final sample comprised 319 gynecological cancer survivors and 1276 women from the general population. After one reminder, 176 survivors (55%) and 521 controls (41%) responded. Some had incomplete responses to most questions and were excluded. In total, 653 responses were included in the analyses; 160 women with and 493 without a history of gynecological cancer. Mean age was 58 and 57, respectively. The mean follow-up time after cancer treatment was 12 years (SD 2.6; range 8-17). More detailed information on the study population is provided elsewhere [16-18].

### Socioeconomic condition

The Socioeconomic Condition Index (SCI) [17] is a modification of the Living Condition Index [19], based on the scores on education, employment, income, ability to pay bills, self- perceived health, and satisfaction with the number of close friends. Education: < 10 years = 0;

10-12 years = 2; 13-15 years = 3; > 15 years = 4. Employment status: unemployed = 0; part- time job = 2; full-time job = 4. The unemployed group included homemakers, students, retired, and women with a disability pension. Annual household income: < 12.500 € = 0;

12.500 - 37.500 € = 2; 37.501-62.500 € = 3; > 62.500 € = 4. Ability to pay bills: never problems = 2; problems = 0. Self-perceived health: very poor = 0; poor = 1; moderate = 2; good = 3; very good = 4. Satisfaction with number of close friends: satisfied with number of close friends = 2; not satisfied with number of close friends = 0.

The summary scores of the SCI range from 0 to 20 and were categorized into quartiles; poor (score ≤ 24 percentile), average to poor (score 25-49 percentile), average to good (score 50-74 percentile), and good (score ≥ 75 percentile) SCI.

### Number of pain sites

A body outline diagram was divided into 30 different areas; 15 on each side of the body, enabling the respondents to locate pain that they had experienced during the past week. As most studies apply an upper limit of 7-10 NPS [8-12,15,20,21], we localized pain to eight body regions: head; neck; chest/stomach; lower abdomen/pelvis/hips; back/buttock; thigh/knees; legs/feet; arms/hands. The marked regions of pain were summarized into NPS (range 0-8).

### Variable specification

The questionnaire also contained questions on potential confounding variables, such as age, marital status (single, married/co-habitant), weight and height (calculation of body mass index, BMI; kg/m2), smoking (yes, previous, never), and co-morbidity. Co-morbidity was measured as diseases/conditions over the past 12 months prior study, with yes/no responses. All questions were assessed by the respondent herself.

### Statistical analyses

All questionnaires were scanned. Consistency analyses were run and corrected for appropriate variables. All analyses were carried out with SPSS version 17.0, applying Chi-square test and forward stepwise logistic regression to examine determinants associated to NPS. Outcome measures were adjusted odds ratios (aOR) with 95% confidence interval (CI). We have used p ≤ 0.05 as level of statistical significance. All reported *p*-values are two-sided.

### Ethics

The study was approved by the Regional Committee for Medical Research Ethics, Mid- Norway, the Norwegian Data Inspectorate, and The National Department of Health and Social Affairs, Norway. All respondents gave informed written consent.

## Results

There were only minor differences in the prevalence of women reporting 0, 1, 2, 3, and 4 or more pain sites (20.2%, 21.1%, 20.2%, 15.8%, and 22.7%, respectively). Pain in the neck was most prevalent (49.9%) followed by pain in the back/buttock (45.2%), lower abdomen/pelvis/hips (35.4%), and thighs/knees (33.8%) (Table 1).

**Table 1:** Pain site responses and total number of pain sites (NPS)

Pain site	N = 653	%
Head	148	22.7

Neck	326	49.9

Chest/stomach	91	13.9

Low abdom/pelvis/hips	231	35.4

Back/buttock	295	45.2

Thigh/knees	221	33.8

Legs/feet	162	24.8

Arms/hands	166	25.4

No. of pain sites (NPS)		

0	132	20.2

1	138	21.1

2	132	20.2

3	103	15.8

4	73	11.2

5	43	6.6

6	23	3.5

7	9	1.4

8	0	0

For the remaining analyses, we categorized NPS as 0, 1-2, 3, and 4-7 pain sites. As displayed in Table 2 age, smoking, and satisfaction with number of close friends were equally distributed among the NPS groups. More women reporting 3 NPS had high BMI. However, in all major factors the differences in the distribution within the NPS groups were found between women reporting 4-7 NPS and those reporting 3 or less NPS. Women with 4-7 NPS were more often single, had lower education, were more often unemployed, had lower income, more problems paying their bills, and poorer general health (Table 2). The SCI summarizes the differences in education, employment status, income, ability to pay bills, self-perceived health, and satisfaction with number of close friends. In total, women with the lowest SCI had the highest NPS (*p *< 0.001).

**Table 2:** Study population characteristics by number of pain sites (NPS)

		Number of pain sites					
	**N**	**0**	**1-2**	**3**	**4-7**	**%**	*P*-value*

	**653**	**20.2**	**41.3**	**15.8**	**22.7**	**100**	

Age							*P *< 0.55

30-49	168	17.9	44.6	18.5	19.0	100	

50-59	188	18.6	41.0	16.5	23.9	100	

60-75	297	22.6	39.7	13.8	23.9	100	

Marital status							*P *< 0.04

Single	152	21.7	32.9	15.1	30.3	100	

Married/cohabit.	501	19.8	43.9	16.0	20.4	100	

BMI (kg/m^2^)							*P *< 0.03

< 25	324	24.7	40.7	11.7	22.8	100	

25-30	241	17.4	40.2	19.5	22.8	100	

> 30	88	11.4	46.6	20.5	21.6	100	

Smoking							*P *< 0.16

Yes	185	15.1	39.5	15.7	29.7	100	

Previous	227	22.5	42.3	15.4	19.8	100	

Never	241	22.0	41.9	16.2	19.9	100	

Education							*P *< 0.10

< 10 yrs	200	16.5	41.0	15.5	27.0	100	

10-12 yrs	186	18.3	38.2	16.1	27.4	100	

13-15 yrs	140	24.3	41.4	15.7	18.6	100	

> 15 yrs	127	24.4	46.5	15.7	13.4	100	

Employment status							*P *< 0.01

Unemployed	326	17.8	35.9	14.7	31.6	100	

Part-time	84	21.4	42.9	16.7	19.0	100	

Full-time	243	23.0	48.1	16.9	11.9	100	

Income (Euro)							*P *< 0.01

< 12.500	33	15.2	45.5	12.1	27.3	100	

12.500-37.500	186	18.8	32.8	15.1	33.3	100	

37.501-62.500	193	19.7	42.5	16.1	21.8	100	

> 62.500	241	22.4	46.5	16.6	14.5	100	

Problem paying bills							*P *< 0.01

Never	533	22.0	42.8	14.4	20.8	100	

Sometimes/often	120	12.5	35.0	21.7	30.8	100	

Satisfied no. friends							*P *< 0.70

Yes	536	20.1	42.4	15.5	22.0	100	

No	117	20.5	36.8	17.1	25.6	100	

General health							*P *< 0.001

Poor	119	7.6	18.5	21.0	52.9	100	

Moderate	158	7.6	44.3	19.0	29.1	100	

Good	176	18.2	48.9	19.9	13.1	100	

Very good	200	39.5	46.0	6.5	8.0	100	

SCI							*P *< 0.001

≤ 24	140	15.0	34.3	15.0	35.7	100	

25-49	170	18.8	35.3	18.2	27.6	100	

50-74	167	18.0	46.7	14.4	21.0	100	

≥ 75	176	27.8	47.7	15.3	9.1	100	

*Chi-square test

The SCI quartiles [poor (score ≤ 24 percentile), average to poor (score 25-49 percentile), average to good (score 50-74 percentile), good (score ≥ 75 percentile)] were evenly distributed by BMI and by co-morbidities such as pulmonary, gastrointestinal, kidney/urinary, skin disorders and migraine/headache. Poor/average to poor SCI-score was more frequent in high age, among singles and smokers, as well as among women who had survived gynecological cancer, had cardiovascular disease, hypertension, diabetes, and musculoskeletal, psychiatric and sleeping disorder (Table 3).

**Table 3:** Demographic factors and co-morbidity by Socioeconomic Condition Index (SCI)

		SCI (quartiles)					
	**N**	**≤ 24 Poor**	**25-49**	**50-74**	**≥ 75 Good**	**%**	*P*-value*

	**653**	**21.4**	**26.0**	**25.6**	**27.0**	**100**	

Age							*P *< 0.001

30-49	168	10.7	16.7	29.2	43.5	100	

50-59	188	8.5	24.5	24.5	42.6	100	

60-75	297	35.7	23.2	24.2	7.7	100	

Marital status							*P *< 0.001

Single	152	36.2	34.2	16.4	13.2	100	

Married/cohabit.	501	17.0	23.6	28.3	31.1	100	

BMI (kg/m^2^)							*P *< 0.22

< 25	324	20.1	22.8	28.4	28.7	100	

25-30	241	21.6	28.2	22.8	27.4	100	

> 30	88	26.1	31.8	22.7	19.3	100	

Smoking							*P *< 0.001

Yes	185	34.1	27.6	20.5	17.8	100	

Previous	227	16.7	23.3	28.6	31.3	100	

Never	241	16.2	27.4	26.6	29.9	100	

History of gyn. cancer							*P *< 0.02

Yes	160	26.9	31.3	22.5	19.4	100	

No	493	19.7	24.3	26.6	29.4	100	

Cardiovascular dis.							*P *< 0.04

Yes	28	39.3	25.0	28.6	7.1	100	

No	625	20.6	26.1	25.4	27.8	100	

Hypertension							*P *< 0.001

Yes	142	29.6	31.7	21.8	16.9	100	

No	511	19.2	24.5	26.6	29.7	100	

Diabetes							*P *< 0.05

Yes	25	36.0	40.0	8.0	16.0	100	

No	628	20.9	25.5	26.3	27.4	100	

Migraine/headache							*P *< 0.40

Yes	243	18.1	28.4	26.7	26.7	100	

No	410	23.4	24.6	24.9	27.1	100	

Musculoskeletal dis.							*P *< 0.001

Yes	148	25.0	37.8	23.0	14.2	100	

No	505	20.4	22.6	26.3	30.7	100	

Psychiatric dis.							*P *< 0.001

Yes	72	34.7	22.2	29.2	13.9	100	

No	581	19.8	26.5	25.1	28.6	100	

Sleeping disorder							*P *< 0.001

Yes	185	27.0	31.9	25.4	15.7	100	

No	468	19.2	23.7	25.6	31.4	100	

*Chi square test

Variables such as SCI (Table 2) and co-factors (Table 3) that were predictors (*p *< 0.10) of NPS in univariate analyses entered forward stepwise logistic regression analyses. Three models were tested: model A (1-2/0 NPS), model B (3/0 NPS), and model C (4-7/0 NPS), with the no-pain-sites group as reference. In all models we adjusted for co-morbidity. Being a gynecological cancer survivor was not associated with NPS in any model. A significant association was found for increasing BMI and NPS in all three models, with no difference between obese and overweight women. Age below 60 years was associated to 3 or more NPS (models B and C) with no difference between the age-groups 30-49 and 50-59 years. A significant association by decreasing SCI and 4-7 NPS was found in model C, but not in model A or B. Although aOR in the lowest SCI quartiles was 4.2 (95% CI: 1.3-13.5) for the 3/0 NPS group (model B), the strongest association between SCI and NPS was found for the lowest quartile of SCI in model C (aOR 16.9; 95% CI: 4.6-61.7) (Table 4). There was no effect modification between any of the significant variables and co-morbidity in any model.

**Table 4:** Predictors of number of pain sites (NPS)

	Model A*	Model B**	Model C***
NPS analyzed	1-2/0	3/0	4-7/0

N controls	132	132	132

N cases	270	103	148

Adjusted odds ratio (aOR)/ Variables	aOR (95% CI)	aOR (95% CI)	aOR (95% CI)

Age			

30-49	1.4 (0.7-2.6)	5.5 (1.9-15.8)	2.9 (0.99-8.5)

50-59	1.5 (0.8-2.8)	4.3 (1.6-11.4)	4.5 (1.6-12.1)

60-75	1.0 (ref.)	1.0 (ref.)	1.0 (ref.)

BMI (kg/m2)			

< 25	1.0 (ref.)	1.0 (ref.)	1.0 (ref.)

25-30	1.5 (0.92-2.4)	3.3 (1.6-6.8)	2.3 (1.0-5.2)

> 30	2.7 (1.2-5.9)	5.4 (1.8-16.5)	1.7 (0.5-5.8)

History of gyn. cancer			

Yes	0.97 (0.6-1.7)	1.4 (0.7-3.1)	1.3 (0.6-3.1)

No	1.0 (ref.)	1.0 (ref.)	1.0 (ref.)

SCI			

≤ 24	1.7 (0.8-3.7)	4.2 (1.3-13.5)	16.9 (4.6-61.7)

25-49	1.1 (0.6-2.3)	1.5 (0.5-4.0)	6.6 (2.0-21.7)

50-74	1.5 (0.8-2.8)	2.1 (0.9-5.2)	6.6 (2.1-20.5)

≥ 75	1.0 (ref.)	1.0 (ref.)	1.0 (ref.)

* Adjusted for musculoskeletal disorders and migraine/headache

**Adjusted for musculoskeletal disorders, migraine/headache, and sleeping disorders

*** Adjusted for musculoskeletal disorders, migraine/headache, sleeping and psychiatric disorders

## Discussion

Major differences in the socioeconomic conditions, measured by SCI, were found between women reporting 4 or more NPS and those reporting 3 or less NPS. The socioeconomic conditions are fairly equal for women reporting 0, 1-2 or 3 NPS (Table 2), with employment status corresponding to women in the general Norwegian population [22]. More women with

4-7 NPS, on the other hand, live under the poorest socioeconomic conditions (Table 2). We did not find a clear socioeconomic gradient in NPS, but a threshold when socioeconomic determinants are associated to NPS. After adjustment for co-factors (Table 3) the strongest association between SCI and NPS was found for women with the lowest SCI scores (Table 4). The association in the other groups is rather modest.

The relationship between low socioeconomic conditions and high NPS could be explained by determinants of social position. Although this relationship has been found for some marginalized groups, there is limited scientific evidence for such associations [23]. On the contrary, components of social position, like material circumstances, lifestyle, and psychosocial factors, have been found to increasingly determine health outcomes. The psychosocial perspective proposes that impaired health is a consequence of long-term stress. Lack of control [24] and relative deprivation [25] may represent the key elements of this association, as both phenomena are related to the lower levels of the social hierarchy in modern societies. Adverse psychosocial environment and low job control [1] as well as experiences of being belittled, lack of social support, and economic hardship [26] is associated with poor self-rated health. Women in deprived socioeconomic positions may experience constant stress due to such unfavorable factors, affecting an imbalance in their hormonal and immune systems [27], leading to pain conditions. Within this context we explain the significant association between low socioeconomic conditions and NPS.

Although a relationship between living in socioeconomic deprived areas and widespread pain has been demonstrated earlier [4], the present study reveals that there is a strong relationship between low socioeconomic status and high NPS. In most studies examining NPS, NPS is treated as a co-factor, with chronic pain [8,11] or disability [21] as outcome variables. In only one study was NPS treated as the dependent variable [12], indicating an association between three components of socioeconomic condition (education, marital and employment status) and NPS in both sexes. However, in that particular study adjustment for co-morbidity was not performed. After adjustment for co-morbidity, we found a strong association between poor

SCI (lowest quartile) and 4 or more NPS, indicating that there is a threshold for most determinants on NPS. In a wealthy country as Norway, women with an average socioeconomic position (≥ 25 and < 75 percentile of SCI) hardly report any higher NPS than women in the best socioeconomic position (≥ 75 percentile of SCI).

Unfortunately, disadvantaged and less assertive women may lack sufficient resources to perform as a credible patient within a normative, biomedical frame of reference. According to Werner and Malterud [28], Norwegian women with chronic pain exert themselves extensively in order to appear as what they hope is "just right" during medical encounters, i.e. substantial effort from the patient's side is required to get access to health care benefits. Health care professionals should be extra attentive to subtle and unarticulated ill-health symptoms of women living in the lowest socioeconomic position to try and reduce the persistent social inequalities in health outcomes [1,2].

As reported in other Scandinavian studies [7,12,13], we found the highest NPS among women below 60 years of age (Table 4). In accordance with Kamaleri et al. [12], we found a weak association between NPS and overweight, whereas smoking and being a gynecological cancer survivor was not associated with NPS in any model. Traumas as war [20] or frightening accidents [29] have been associated with NPS later in life, but surviving cancer without recurrence seems to be very different from surviving other traumas.

The sample size (N = 653) and the high completeness of reported data, including pain areas on the body chart, are considered strengths of the present study. The prevalence of women reporting no, one, two, three, or 4-7 pain sites (Table 1) is similar to what is reported in another Norwegian study [12], supporting the external validity of the study. We consider the use of SCI as strength of the study, and we avoid the problems of co-linearity in multivariate models applying a single outcome for socioeconomic conditions. One limitation of the present study is the cross-sectional design. We cannot draw strict conclusions on causality as the relationship between socioeconomic conditions and NPS is very complex and interactive. Another limitation is the relatively modest response rate. However, the response-rate among gynecological cancer survivors and their controls selected at random from the general population, 55% and 41%, respectively, is considered high, related to comparable studies [4,13,30]. There was no skewed distribution between respondents and non-respondents among survivors and women selected at random from the general population regarding age (quartiles) or marital status (married/single) (data not shown).

## Conclusion

After adjustment for age, BMI and co-morbidity, we found a strong association between low SCI score and four or more NPS, indicating that there is a threshold in the NPS count for when socioeconomic determinants are associated to NPS in women.

## Abbreviations

BMI: Body mass index; SCI: Socioeconomic condition index; NPS: Number of pain sites

## Competing interests

Non-financial competing interests

## Authors' contributions

Toril Rannestad (TR), Finn Egil Skjeldestad (FES). Both authors designed the study, organized the questionnaire. The pilot study was undertaken by TR. Administration of adresses lists, mailing, and reminders were done by a professional market company (Sentio AS, Trondheim, Norway). Consistency analysis of raw data, appropriate corrections, organizing the data file, and analysis were done by FES. Both authors contributed to the interpretation of data and writing of the manuscript. Both authors read and approved the final manuscript.

## Funding

The study was funded by a grant from SINTEF Health, Trondheim, Norway, and by a grant from the Sør-Trøndelag University College, Faculty of Nursing, Trondheim, Norway.

## Pre-publication history

The pre-publication history for this paper can be accessed here:

http://www.biomedcentral.com/1472-6874/12/7/prepub
